# Ettore Beghi (1947–2022): A farewell

**DOI:** 10.1002/epi4.12668

**Published:** 2022-11-10

**Authors:** Aristea S. Galanopoulou, Dong Zhou

**Affiliations:** ^1^ Neurology & Neuroscience Albert Einstein College of Medicine Bronx New York USA; ^2^ Department of Neurology Sichuan University West China Hospital Chengdu China


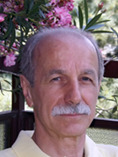


It is with great sorrow that the editors of *Epilepsia Open* wish to bid farewell to one of our most valued associate editors, trusted friends, and colleagues, Ettore Beghi, who passed away on October 10, 2022.

Ettore Beghi had been invited to our editorial board in 2016, when *Epilepsia Open* was founded. He had since been tirelessly working towards enriching and improving the quality of the content published in our journal, as associate editor, always offering his advice, encouragement, and precious ideas to raise the journal to a highly successful course. Ettore brought his unique expertise, knowledge, and interest in epidemiology, quickly attracting a large number of articles reporting on the epidemiology of seizures, epilepsies, or their comorbidities across the globe. He would always offer a gracious personal message to the authors of the manuscripts he handled. Until the very last time when he was able to participate in our editorial virtual meetings, Ettore was always active, smiling, collegial, and prolific with ideas.

Ettore Beghi was born in Milan, Italy, on August 15, 1947. He studied medicine (1972) and neurology (1976) in Milan, obtained a Master of pharmacologic sciences research at the *Istituto Mario Negri* in Milan (1981), and served as a research fellow in the department of medical statistics and epidemiology at Mayo Clinic in Rochester Minnesota (1982–1983). He had been the head of the laboratory of neurological disorders of the *Istituto di Ricerche Farmacologiche Mario Negri* and a contract professor of neuroepidemiology at the University of Milan. His medical and research career was notable for seminal studies in the epidemiology of epilepsy but also extended to other neurological disorders, such as peripheral neuropathy, amyotrophic lateral sclerosis (ALS), Parkinson's disease, headache, and strokes. He authored 642 manuscripts.

Ettore Beghi's scientific and academic achievements have been recognized through the Ambassador for Epilepsy award of the International League Against Epilepsy (ILAE). He had served on numerous committees as a chair or member, including chairing the ILAE commission on the epidemiology of epilepsy and the neuroepidemiology section of the American Academy of Neurology. He had served on numerous editorial boards, including the editorial boards of *Epilepsia Open* and *Epilepsia* *.* Ettore was a coordinator of the European ALS registry and the Italian Epilepsy Study Group and a pioneer of multicenter neuroepidemiological studies in Italy. He was a valued teacher and mentor to many.

Over the last 8 years, our journal had been fortunate to share his enthusiasm for epilepsy research and excellence, humanity, and dignity that was apparent in every interaction with Ettore. We are grateful to him and his family for sharing him with us, and we express our most heartfelt condolences to his wife Maria Lidia, his children Massimiliano, Emanuele, and Nadia, and his grandchildren. He will be missed, but his memory, contributions, and accomplishments will live on.

